# Incorporation of Biochar to Improve Mechanical, Thermal and Electrical Properties of Polymer Composites

**DOI:** 10.3390/polym13162663

**Published:** 2021-08-10

**Authors:** Chinmoyee Das, Sandeep Tamrakar, Alper Kiziltas, Xinfeng Xie

**Affiliations:** 1 College of Forest Resources and Environmental Science, Michigan Technological University, Houghton, MI 49931, USA; cdas2@mtu.edu; 2 Research and Innovation Center, Ford Motor Company, 2101 Village Rd, Dearborn, MI 48124, USA; stamraka@ford.com (S.T.); akizilt1@ford.com (A.K.)

**Keywords:** biochar, carbonization, polymer composites, sustainability, composite properties

## Abstract

The strive for utilization of green fillers in polymer composite has increased focus on application of natural biomass-based fillers. Biochar has garnered a lot of attention as a filler material and has the potential to replace conventionally used inorganic mineral fillers. Biochar is a carbon rich product obtained from thermochemical conversion of biomass in nitrogen environment. In this review, current studies dealing with incorporation of biochar in polymer matrices as a reinforcement and conductive filler were addressed. Each study mentioned here is nuanced, while addressing the same goal of utilization of biochar as a filler. In this review paper, an in-depth analysis of biochar and its structure is presented. The paper explored the various methods employed in fabrication of the biocomposites. A thorough review on the effect of addition of biochar on the overall composite properties showed immense promise in improving the overall composite properties. An analysis of the possible knowledge gaps was also done, and improvements were suggested. Through this study we tried to present the status of application of biochar as a filler material and its potential future applications.

## 1. Introduction

### 1.1. Biochar

Biochar is the carbon rich solid material that is left after the thermochemical conversion of biomass in an oxygen limited environment [1]. Features like low density and ecological sustainability make it an attractive replacement for inorganic fillers [2]. Biochar can be obtained from biomass by various thermal decomposition methods like pyrolysis, combustion, gasification and liquefaction [3]. Precisely, biochar is produced by a method called carbonization. Carbonization and pyrolysis share the same fundamental principle where the raw material undergoes thermal decomposition in an inert atmosphere. However, the process of carbonization is undertaken at a much slower pace and heating rate in order to enhance carbon yield of the process. Several factors, such as feedstock type, carbonization process, pyrolysis temperature, heating rate, and residence time can influence the physiochemical properties of biochar [3]. Biochar is produced using various lignocellulosic resources that can be incorporated into composites as fillers to enhance various properties of composites. Pyrolysis is the process of carbonization of raw material in an oxygen free environment to obtain carbon rich product. Behazin et al. (2018) [4] used miscanthus grass as the raw material for biochar. Waste materials generated from processing can be used as a raw material. Waste materials like pine wood, date palm, oil palm empty fruit bunch and rice husk, cashew nut shell have been used to produce biochar to be used as reinforcing filler in polymer matrices [5,6,7,8]. Utilization of processing waste materials as biochar feedstock closes the loops of production making it a circular and sustainable process [9]. S. Zhang, Yao, Zhang, & Sheng, 2018 [10] used bamboo to produce biochar, as filler in the composites. Bartoli et al. (2020) [11] used olive pruning as raw material for production of biochar used in manufacture of biochar filled epoxy composites. Idrees, Jeelani, and Rangari (2018) [12] produced biochar out of packaging waste consisting primarily of starch-based packing peanuts. Other organic materials like sewage sludge and bird litter have been reportedly implemented for manufacturing biochar [13,14]. The feedstock of biochar is fairly versatile, and the properties can be tailored by fine tuning the carbonization process.

The morphological structure and properties of biochar have made its implementation possible in diverse arenas. The excellent adsorption capabilities of biochar make it a great soil conditioning agent and is used to contribute to soil health by restoring trace elements [15]. Biochar can be used as an adsorbent in wastewater treatment systems and has been used in several water filtration systems as well. The hygroscopicity of biochar can be altered by controlling the temperature of carbonization and the resultant biochar can be used as a soil additive to improve moisture holding capacity of the soil. A major sector that is looking into the application of biochar is the plastic composite industry. Polymer composites are multiphase materials reinforced with a filler, resulting in improved mechanical properties due to the synergistic effect of the two [16]. Automobiles are a major contributor to greenhouse gas emissions. As per Environmental Protection Agency (EPA), an average 8887 g of CO_2_ is emitted from 1 gallon of gasoline and 10,180 g of CO_2_ is emitted from 1 gallon of diesel [17]. Annually 4.6 metric tons of CO_2_ is released from a typical passenger vehicle [17]. These emissions have a highly detrimental effect on the global climate scenario. Increased fuel efficiency of vehicles can lead to lower fossil fuel consumption, in turn lower greenhouse gas emissions. To achieve this, the automobile manufacturers have resorted to lightweighting of the vehicles. As per the EPA 2020 Automotive trends report, the heaviest vehicles produced in model year 2020 generate half the amount of CO_2_ compared to what was generated in model year 1978 [18]. The CO_2_ generated for lighter vehicles in 2020 is two thirds of what was generated in 1978, all owing to the massive design changes and advancements undertaken by the automakers [18]. Lightweighting can be achieved by making smaller cars or by material substitution without compromising on the capacity and size [19]. Traditional materials are being replaced by lightweight metals and largely by polymer composites. These composites are generally filled with fillers like talc, glass fibers, calcium carbonate (CaCO_3_), clay nanoparticles [20,21], etc. These polymer composites are lighter than the conventional metals, but the inorganic fillers have a high density making the end products bulky. Biocomposites come into play here. Biocomposites are a category of polymer composites that are biocompatible and/or eco-friendly [22,23,24]. The fillers in biocomposites are biomass derived from plants or animals, however, natural fillers are generally polar leading to interfacial adhesion issues with the fairly non-polar polymer matrices. Biochar is relatively inert compared to unprocessed natural fillers and can serve as filler in polymer composites fortifying the composites and with further calibration, additional properties like electrical conductivity can also be introduced in the composites, as per requirement.

**Figure 1:**
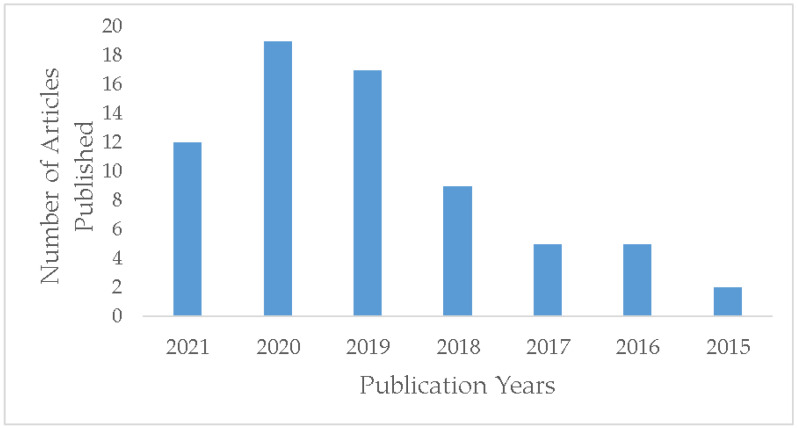
The number of studies based on biochar filled polymer composites published each year between 2015 and 2021. This image is taken from Web of Science (https://www.webofscience.com/, accessed on 15 July 2021). Certain data included herein are derived from Clarivate Web of Science. © Copyright Clarivate 2021. All rights reserved.

As per Web of Science, in the last decade between the years 2015–2021, 70 research articles have been published based on the incorporation of biochar as a filler in polymer composites. The literature search was done using the keywords polymer, biochar filler and composites. A yearly publication statistic is presented in Figure 1. The figure shows a steady growth between the years 2015–2020 in the research done on application of biochar filler in polymer composites. A significant number of articles have been published in the past 6–7 months of 2021 as well. This is indicative of the growing interest towards application of biochar as a composite filler over the years.

In this review paper we present a comprehensive description of the current research on the utilization of biochar as a filler in polymer composites. A description of the different methods and parameters used in these studies are summarized along with a critical analysis of their results and findings. An overall representation of the research including the bottlenecks and possible solutions to them is included in the end of this review. Adhering to the objective of the review paper, the state-of-the-art research in this field is summarized in this review to have an overview of the different approaches and results developed based on these approaches.

### 1.2. Structure of Biochar

The inherent properties of biochar are greatly dependent on the structure. Properties like electrical conductivity can be manipulated greatly by the structure of biochar. Carbon has several allotropes that are crystalline and amorphous. Graphite is a crystalline carbon allotrope with great electrical properties owing to its structure known as graphitic structure. Graphitic structure of material can be characterized by the presence of highly ordered well stacked graphene sheets [25]. Graphitizable biomass has the tendency of obtaining a very highly arranged structure at a very high (>2000 °C) treatment temperature [25]. The biochar structure is composed of ordered crystalline turbostatically arranged crystalline regions and disordered amorphous regions [26], obtained from the intrinsic structure of biomass, predominantly composed of cellulose. The presence of these turbostatically ordered regions contribute to the electrical conductivity of biochar [27]. This isotropic structure results in spaces forming a porous structure [25]. The honeycomb like pores on the surface of biochar result in mechanical interlocking of polymer with the biochar making it a very good reinforcing agent [28].

**Figure 2:**
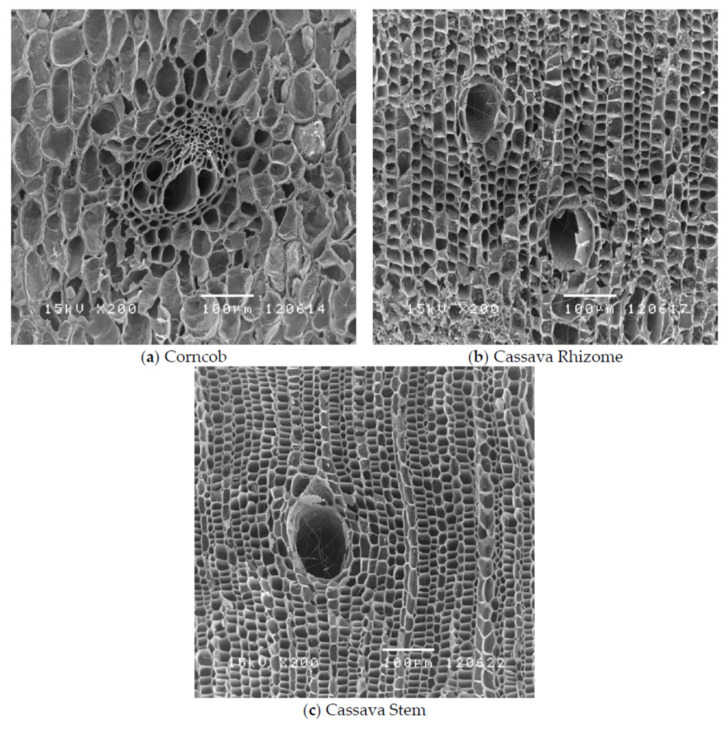
SEM images showing biochar produced from different lignocellulosic feedstocks (**a**) corncob (**b**) cassava rhizome (**c**) cassava stem. Each feedstock source has a distinctive morphology which is retained in the biochar after carbonization. This image is taken from [29] from Applied Sciences Open Access journal MDPI publications.

Figure 2 here shows the honeycomb structure of biochar derived from different feedstocks like corncob, cassava rhizome and cassava stem [29].

## 2. Composite Formation

Polymers like polypropylene, polyethylene, nylon, epoxy, etc., are used in the form of composites filled with inorganic fillers like CaCO_3_, talc, glass fibers and clay nanoparticles have also been used as fillers in several composites [20,21,30]. Carbon based fillers like carbon fibers, carbon nanotubes and carbon black have been used to improve the mechanical properties, along with making the non-conductive matrix electrically conductive [30,31,32]. These conventional fillers can be substituted with biochar to develop light sustainable composites that are cost effective and improve the carbon footprint of users throughout their lifecycle. The fabrication of biochar filled composites is carried out using the commonly used composite fabrication methods like melt extrusion and injection molding. Some of the studies addressed here have used not very commonly used methods like solvent casting, resin curing, etc., [33,34]. The melt processing of polymer takes place in three steps involving melting, shaping and solidification of polymer in the desired shape [32]. Melt extrusion of polymers can be done using a single screw extruder or a twin-screw extruder. Application of heat and pressure results in dispersion of external filler, coloring agents, etc., in the polymer matrix. Melt processing of plastics depends on several polymer characteristics like melting point, melt viscosity, etc. The process can be optimized by controlling the barrel temperature of the extruder and the rotation speed of the screw. In injection molding, which is largely used on an industrial scale, the molten polymer is injected into a mold and casted into the shape of the mold. Solution casting of polymers is based on Stokes law and is achieved by dissolution of the polymer and the additives in a solvent or different solvent and then mixing them together. The mixture is then dried to evaporate the solvent and render the composite [35]. The various fabrication methods and compositions used in the studies done on biochar filled polymer composites are summarized in Table 1.

**Figure 3:**
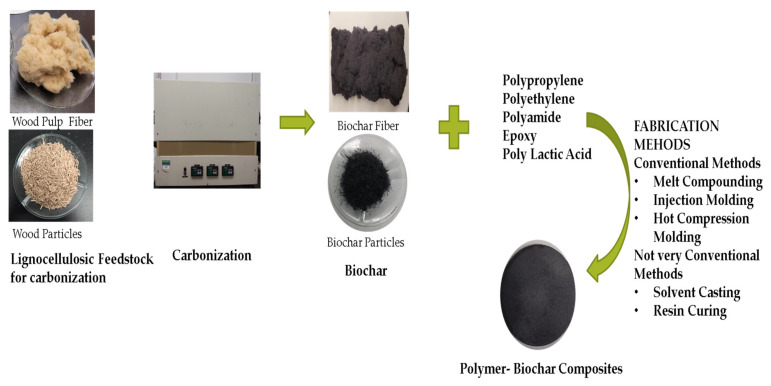
An illustration showing the development of biochar filled polymer composites.

A few studies have reported very interesting and novel methods undertaken in their particular studies. Li et al. (2016) [36] induced a negative charge on their polymer using a high shear mixing technology to obtain segregated biochar network in the composites [48]. This method has been deemed successful in the study. Utilization of intrinsic properties of the polymer to customize biocomposite properties has not been reported anywhere else so far. Ferreira et al. (2019) [41] used commercially available sugarcane bagasse biochar in their study. They chemically treated their filler with a base (NaOH) and acid (HCL) in a leaching process followed by Isopropanol vapor thermal annealing to remove polar groups from the biochar surface for better interfacial adhesion in composites. The method developed by them is quite unique and has been successful in improving interfacial properties of the composites. It can be observed that injection molding and melt compounding methods have been largely used for composite fabrication. These methods have been largely used since melt extrusion and injection molding are practical and more efficient on a commercial scale. They are equipped to process a large amount of material with ease making the overall process efficient. Solvent casting and resin curing methods work on a smaller scale, however, they are not preferred commercially as scaling up of these methods to a commercial level will not be as efficient as injection molding and/or melt compounding. Another factor to look into in the fabrication process is addition of external additives as compatibilizers. Maleic anhydride grafted polypropylene (MAPP) is the most commonly used additive reported in most of the studies using a polypropylene matrix. Figure 3 presents an overall overview of the steps involved in fabrication of polymer-biochar biocomposites.

## 3. Composite Properties

### 3.1. Mechanical Properties

The mechanical properties of the composites are determined by testing the composites for tensile strength, tensile modulus, flexural strength and modulus, impact strength, elongation on break. There are several factors like filler loading rate, interfacial adhesion, presence of compatibilizer, distribution of filler, particle size, carbonization temperature, etc., that play an important role in determining the mechanical properties of biochar filled polymer composites.

The quality of a composite depends a lot on the amount of filler incorporated in it. Das, Bhattacharyya et al. (2016) [5] designed a study with different biochar loading rates and characterized their composites for tensile strength and flexural strength. The tensile strength results show that the composites with 15% biochar exhibited higher stress yield compared to higher loading rates [5]. The composites with higher quantities of biochar (25, 30 and 35%) fractured earlier showing semi-brittle behavior under tensile stress [49]. The tensile strength of the composites however did not undergo a drastic change and the values were close to the tensile strength value of polypropylene. The tensile modulus values, however, showed a positive trend with increase in the amount of filler. The modulus value for the composite with 35% biochar was recorded to be 3.82 GPa [5]. This property is attributed to the high surface area of biochar developed as a result of carbonization at 900 °C, enabling better stress transfer between polypropylene and biochar [5]. Stiffness of polypropylene increased steadily on addition of biochar. The increased stiffness resulted in lower percentage elongation for composites with higher biochar concentration. The composites with 15% biochar reported the best elongation. Both flexural strength and modulus of the composites showed an improvement with the addition of biochar. The factors affecting flexural strength of a composite are particle dispersion, wetting and infiltration of polymer in filler particles [5]. High surface area of biochar resulted in physical/mechanical interlocking between polypropylene and biochar, as molten polypropylene entered the pores present on biochar surface. This interlocking caused a reinforcing effect in neat polypropylene and facilitated stress transfer [5] reinforcing the composite. Similarly, Khan et al. (2017) [34] observed transition of a brittle epoxy into a ductile matrix on addition of any carbon filler in small amounts. The addition of carbon fillers enhanced the mechanical properties of the composites [34]. However, an increase in filler content was associated with the decline in tensile strength and elongation of the composite, this phenomenon is also observed in the study designed by Das, Bhattacharyya et al. (2016) [5]. Improved mechanical properties of composites is attributed to the stress transfer mechanism between the filler and matrix obstructing failure. It is stated that addition of biochar also improved the cross linking of epoxy matrix blocking the molecular motion of the polymer adding to the strength of the matrix [50,51]. Enhanced thermal conductivity of the composite due to the higher thermal conductivity of filler leads to lower concentration or faster diffusion of heat generated due to plastic deformation at a given section improving the ability to tolerate higher strain before reaching the glass transition temperature (Tg) [52]. The Ultimate Tensile Strength (UTS) of the composites improved at lower filler loading and showed drastic decline with increasing filler loading rate, this trend was also consistent with the load bearing capacity of the composite. The deterioration in the load bearing capacity of the composite was due to the transition of behavior of matrix from plastic to semi-brittle [49]. With higher filler concentration the stacking and cross linking of polymer increases leading to a brittle behavior [53]. The studies discussed here indicate that addition of biochar definitely improves the mechanical properties of composites, irrespective of the concentration. The crosslinking between the matrix and filler is attributed to this enhancement in both the studies. It is also observed that with increasing biochar concentration the tensile strength and percentage elongation is reduced, due to the increased stiffness on filler incorporation. From these results it can be understood that concentration of filler dictates the mechanical properties, and an optimal filler concentration is important for good mechanical properties of composites.

**Figure 4:**
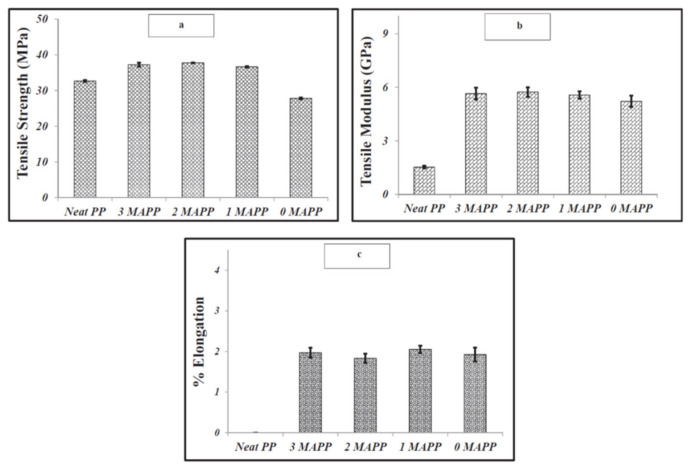
The figures here show the mechanical properties of biochar filled polypropylene composites (**a**) Tensile strength, (**b**) tensile modulus and (**c**) percentage elongation. Figure is taken from [42] with permission to reuse from Elsevier Publications 2016.

Interfacial adhesion is a phenomenon that is observed when two or more components are mixed with each other [54]. Good interfacial adhesion usually means better dispersion of filler in the matrix, leading to improved composite properties. Optimal interfacial adhesion is achieved when there is no repulsion between the components of a composite. Polarity differences between the matrix and the filler can lead to repulsion between them causing poor interfacial adhesion. Additives like compatibilizers can be included in the composition to improve the compatibility between the matrix and the polymer. A very commonly used compatibilizer is maleic anhydride grafted polypropylene (MAPP), that is usually used in composites developed using polypropylene matrix. The presence of MAPP reinforces the interfacial adhesion in the composites by forming a bond between the hydroxyl groups of the compatibilizer and polypropylene matrix [28]. Composites can also be developed using fillers that can be natural or inorganic in nature, such composites are known as hybrid composites. These composites are developed with an intention to study the effect of filler(s) individually and collectively on composite properties. Wood is predominantly used as an additional filler with biochar in the studies mentioned here. Das, Bhattacharyya and Sarmah (2016) [42] developed hybrid composites filled with wood and 24% biochar along with compatibilizer MAPP (0–3%) in a polypropylene matrix. They reported improved tensile strength in composites with increasing compatibilizer content. The highest value for tensile strength was obtained for composite samples with 3% MAPP and the lowest for composites with no compatibilizer [42]. Even at 1% compatibilizer loading rate, a significant improvement in composite properties was observed, indicating synergistic effect of MAPP on composite mechanical properties. The tensile strength of composites mainly depends on the quality of bonding that includes chemical, physical, adsorption, electrostatic forces, etc., between the polymeric matrix, the filler and the additives in the composite [55]. The tensile modulus values for the composites were much higher when compared to neat polypropylene. The statistical analysis of the tensile modulus values for the composites with and without MAPP did not show much difference indicating improvement in properties irrelevant of MAPP [42]. The percentage elongation values for the composites were low due to the increased brittleness introduced upon adding biochar [56]. The flexural strength and modulus values for the composite were reported to be significantly higher compared to neat polypropylene; this improvement is attributed to addition of wood and biochar that improved the flexural properties. The flexural strength of a composite depends on factors like particle dispersion, wetting and infiltration of molten polymer in the filler particles [42]. The interlocking between the polymer matrix and filler particles resulted in the improved flexural strength of the composites. Impact analysis of a composite gives an idea of the interfacial adhesion between the polymer and the filler. Wambua, Ivens, and Verpoest (2003) [57] stated that composites with poor interfacial adhesion show fiber pull out and expend more energy while composites with good interfacial adhesion show fiber fracture and dissipate less energy [57]. Therefore, a composite with good interfacial adhesion will have lower values for impact strength [42]. The impact strength results for the composites indicate good interfacial adhesion between the polymer and filler; this phenomenon is not affected by the presence and amount of MAPP. The micro-hardness of the biocomposites was analyzed using Vickers hardness test. The test results showed improved hardness in the composites in comparison to neat polypropylene indicating the positive impact of addition of biochar filler. The composites without MAPP had lower hardness value in comparison to the composites with MAPP. This is attributed to better adhesion between wood and polypropylene specifically due to the presence of MAPP. The lack of functional groups on the surface of biochar resulted in no bonding between the biochar filler and MAPP [42]. As a result, the effect of MAPP was more pronounced in regions of the composite with a higher wood filler concentration, respectively. The Figure 4 below indicates that the mechanical properties did not express a drastic change with the presence of MAPP. This indicates compatibilization is synergistic to composite, however, does not necessarily have a significant impact on the composite properties in this study [42].

In a similar study, Ikram et al. (2016) [28] reported the tensile strength of the composites having MAPP to be better than those not containing the compatibilizer. Mechanical interlocking between the PP matrix and biochar filler facilitated by the high surface area of biochar, due to the absence of functional groups on its surface contributed to improved tensile strength. A similar trend was observed for tensile modulus; the presence or absence of wood played a major role in improving the modulus of the composites. Presence of wood improved the tensile modulus of the composite as distance between the particles was reduced improving the rigidity and stiffness of the composite [58]. The samples containing a higher amount of wood and biochar reportedly had improved modulus values [28]. Flexural strength of the composites followed a similar pattern as tensile properties due to the presence of coupling agent MAPP. An important physical process contributing to this trend is the infiltration of polypropylene matrix in the pores present on the biochar surface. Like tensile properties, presence of wood enhanced the flexural properties of the composites. Das, Sarmah, and Bhattacharyya (2016) [59] developed hybrid biocomposites having a polypropylene matrix filled with biochar at different loadings (6, 12, 18, 24 and 30 wt%), wood and coupling agents. The modulus and hardness assessment of each component indicated that neat propylene had the highest load vs. displacement curves exhibiting a plastic behavior compared to wood and biochar [59]. The behavior of biochar was found to be more elastic compared to the behavior of wood due to the presence of C–C covalent bonds [59]. The result for hardness and modulus value for neat PP was reported to be 1.5 GPa and for wood and biochar were reported to be 5.6 and 5 GPa, respectively [59]. The slightly lower modulus value of biochar when compared to wood can be attributed to the increased stiffness caused by degradation of biopolymers like cellulose in the process of carbonization [60]. The indentation of the composites with varying filler loading indicated the influence of individual mechanical properties of wood and biochar on the mechanical properties of the composite at areas near the interface. Incorporation of wood and biochar into the matrix reinforced the hardness of the matrix improving the modulus of the composites. Enhanced reinforcement is a result of mechanical interlocking of polymer with biochar owing to enhanced matrix wettability due to high surface area of biochar [61]. The bulk properties of the composites were predicted using various theoretical models like rule of mixtures, Halpin–Tsai–Nielsen and Verbeek models. To overcome the possible shortcomings of nanoindentation, the composite samples were characterized for Vickers hardness test. The hardness of the composites improved with the increase in biochar content, which was an expected phenomenon. The bulk hardness of the composites was predicted using the rule of mixtures and it was observed that a good correlation was found between the predicted values and the values obtained using Vickers hardness test [59]. Similarly, Das, Sarmah, and Bhattacharyya (2016) [59] stated that a strong positive correlation was found between the predictive models (rule of mixtures, Haplin–Tsai–Nielsen and Verbeek models) and experimental values for flexural moduli of the composites, a moderate correlation was found between the predictive models and the bulk experimental tensile moduli values. This study was a novel approach taken by the researchers to study the individual components as well as the composites. It was concluded that prediction of overall composite properties requires determination of other factors like aspect ratio and filler orientation which cannot be assessed with nanoindentation, hence the indentation study worked better for individual components rather than the whole composite. Behazin, Misra, and Mohanty (2017a) [37], designed an experimental study to investigate the effect of biocarbon particle size, type of compatibilizer and concentration of compatibilizer on polypropylene/biochar composite properties. The results for mechanical properties were reported for matrix alone and for biocomposites as well. The matrix that was composed of polypropylene hardened by POE showed higher stiffness and lower impact toughness when MAPP was used as compatibilizer instead of MAPE [37]. Similar trends are also observed for tensile properties. On an average the addition of compatibilizer without a filler was observed to be detrimental to the properties of the composites. In biocomposites, filler particle size, compatibilizer type and concentration had an impact on the composite stiffness and impact properties. It was observed that there was a steep increase in the Young’s modulus of the composites at a concentration of 5 wt% of MAPP [37]. The impact of factor “compatibilizer type” was the most pronounced compared to the other two factors biocarbon particle size and compatibilizer type. Improvement in mechanical properties was seen for larger particle size in the presence of MAPP. The particle size of filler was the next most influencing factor after compatibilizer type in this study. It was observed the particle size between 106 and 125 µm gave the best results for mechanical properties of the composites [37]. The studies designed using compatibilizer report the improvement of composite properties in the presence of both filler and compatibilizer. However, interaction between compatibilizer and biochar filler is limited as biochar produced at higher temperatures lacks functional groups, preventing interaction between the compatibilizer and filler [26,39]. The improvement in mechanical properties was largely due to the mechanical interlocking between the matrix and biochar and was not quite related to the presence of compatibilizer. However, presence of compatibilizer did strengthen the matrix adding to the enhancement of mechanical properties of the composite.

**Figure 5:**
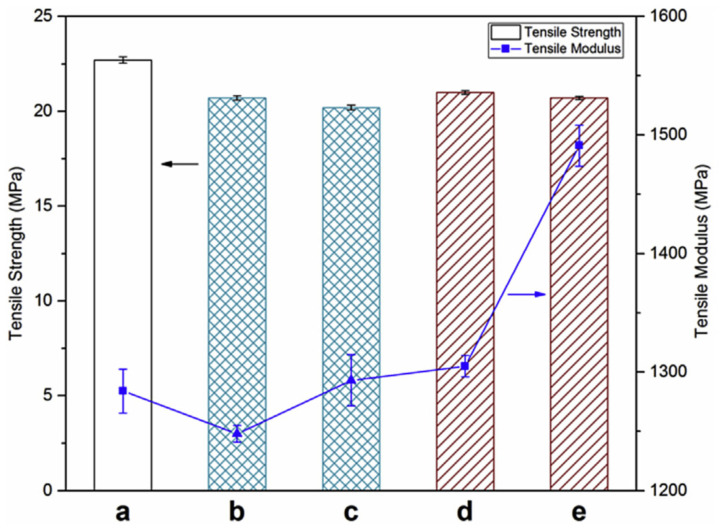
The tensile properties of biochar filled composites, (**a**) neat polymer, (**b**,**c**) LtBioC filled composites and (**d**,**e**) HtBioC filled composites. The tensile properties of composites filled with HtBioC were shown to be better than neat polymer and composites filled with LtBioC. Figure is taken from [38] with permission to reuse from Elsevier publications 2017.

The properties of biochar filled composites can be altered by the carbonization temperature of the filler. The carbonization temperature of filler can alter the surface area and the functionality of biochar greatly. Several studies have been designed to study the effect of temperature on composite properties. Behazin, Misra, and Mohanty (2017) [38] studied the effect of miscanthus biochar carbonized at different temperatures on the mechanical properties of biochar filled biocomposites. The tensile strength of the composites was reported to be lower than toughened polypropylene matrix. The tensile modulus values on the other hand were higher for the composites having High Temperature Biochar (HtBioC) in comparison to the toughened matrix and composites having Low Temperature Biochar (LtBioC). The biochar used in the composites has higher modulus [62] compared to the polypropylene used in the matrix resulting in composites having higher modulus. The highest value for tensile modulus was seen for biocomposite having 20% HtBioC filler. This property, however, was observed only in composites with 20% filler content, which is due to the encapsulation of hard filler particles with softer phase of the hardening agent polyolefin elastomer (POE) hindering stress transfer to the filler [63]. In the case of composites having 20% biochar in composition, as biochar has a higher ratio the effect of biochar filler modulus on the composite modulus was more prominent. Elongation at break and impact strength of the composite showed a declining trend and had significantly lower values when compared to the hardened matrix. This is the result of the incompatibility of the matrix and filler, predominantly in the case of LtBioC due to the presence of functional groups on its surface [38]. Composites having HtBioC showed higher values for elongation at break in comparison to LtBioC filled composites. The effect of carbonization temperature is quite prominent on mechanical properties as indicated in Figure 5. It can be seen that the tensile properties of composites filled with HtBioC are definitely better than composites filled with LtBioC.

On a similar note Ogunsona, Misra, and Mohanty (2017a) [40] developed their study exploring the effect of biochar carbonization temperature and interfacial adhesion on biochar filled nylon 6 composite properties. A 20% increase in the tensile properties of composites filled with B1 was observed, the increase is attributed to good interfacial adhesion between the polymer and filler facilitated by enhanced wetting of filler. The enhanced interfacial adhesion resulted in efficient stress transfer between the polymer and filler improving the mechanical properties [40]. In composites filled with B2 the increase was of 12.6% and a significant difference was not observed between the value obtained for the composite and neat nylon 6, however, a huge standard deviation value was recorded. This was reported to be a result of improper wetting of the filler by the polymer matrix causing irregular stress transfer, hence the result. A hybrid composite was developed combining B1 and B2 filler in nylon 6 matrix and was characterized. The value of tensile strength of the hybrid composite was a value falling in between the values recoded for B1 and B2. It is stated that the strength value of the hybrid composite was more dependent on B1 as it comprised 50% of the filler content [40]. On incorporation of B1 and B2 an increase of 30% and 26% in the flexural strengths of composites was observed. The flexural strength values follow the similar trend as observed in tensile strength values, the improvement is higher in B1 filled composites compared to B2 filled composites. This difference is again attributed to the interaction between the filler and matrix facilitating transfer of stress and relaxation resulting in better flexural properties [40]. As observed before, in this case too, better interaction between nylon matrix and B1 has resulted in improved flexural properties in the composite. The tensile modulus of the composites also observed an increase compared to neat polymer, however, the improvement was not reflective of the huge difference in modulus values recorded for the biochar B1 and B2 individually [40]. In the case of tensile modulus, the difference in wetting of filler by molten polymer has been reported to have a significant effect on the difference of values [58]. Similar results were obtained for flexural modulus values as well. The impact strength values, however, showed an interesting trend, as composites filled with B2 showed a drastic decrease in impact properties. The decrease was recorded to be almost 32%, while for composites filled with B2 a huge difference was not observed [40]. The lower values of impact strength of B1 filled composites is actually attributed to good interfacial adhesion between polymer and filler as a good adhesion results in transfer of crack energy through the composite and due to absence of voids or deformities the crack energy cannot be dissipated resulting in inferior impact properties [40]. In the case of composites filled with B2, the poor interfacial adhesion between matrix and filler forms many voids in the interface resulting in energy dissipation, lowering of glass transition temperature Tg in these composites also enhances the matrix ability to plastically deform improving the impact properties of the composite. Basically, good interfacial adhesion between the biochar B1 and matrix is the cause of poor impact properties. The elongation at break values reduced on addition of biochar filler. However, the values for B2 filled composites were higher compared to B1 filled composites due to the poor interfacial adhesion, allowing easier flow of polymer chains. This is also supported by the lower Tg value of composite on addition of biochar [40]. The difference in interfacial adhesion results in difference in mechanical properties of the composites. This difference in property can be applied in defining different functions for the composites based on the mechanical performance. B1 filled composites can be used in applications which require higher tensile strength and modulus, similar to applications implementing use of talc filled composites in automobile components [40]. While, B2 filled composites can be applied to use where composites having high impact resistance with moderate strength and modulus values are required [40]. Similarly, Giorcelli et al. (2019) [64] carried out a study to determine the applicability of biochar carbonized at low and high temperature (HT), as a cheap and environmentally friendly filler to improve properties of epoxy polymer. The mechanical properties of the composites showed that incorporation of 1% of biochar filler did not affect the ductility of the polymer much, at loading rates of 2% and 4 wt% of both biochar and biochar (HT) the brittle matrix became ductile improving the elongation of the composite [64]. The tensile strength assessment showed that even at 1 wt% biochar loading, the load bearing capacity of the composite increased to 63%, compared to neat polymer [64]. The improved properties are attributed to the transfer of stress from the matrix to the filler. A phenomenon of cavitation of debonding of polymer on application of stress has been observed [64]. The phenomenon of cavitation and filler pull out is considered the reason for improved mechanical property of the composite [63,64]. Another reason attributed to the enhancement of tensile strength is the crosslinking between the polymer and the filler which is believed to be effective in blocking the molecular motion of the polymer molecules and enhancing the strength of the matrix as well as the composite [47,48]. The Young’s modulus of the composite saw an improvement even at 1 wt% biochar and biochar (HT) loading, the values were enhanced by 33% and at 20% biochar addition it was enhanced by 41% only which is very close to the value achieved using 1% filler loading [64] indicating the concentration of filler is not a significant player in this case. Overall, an improvement in the stiffness of composite was observed on addition of biochar and biochar (HT) filler. Tensile toughness is another property of composite that was analyzed in the study. Tensile toughness is defined as the capacity of the composite to withstand load before breakage or the energy per unit volume needed to break the composite [64]. Addition of 1% of biochar filler did now show much difference, while at 2 wt% filler loading the value for tensile toughness saw 11-fold increase. However, increase in the loading rate of filler did not further make any improvements, rather it deteriorated composite toughness. This is attributed to the fact that a semi-brittle behavior is observed due to uneven distribution of filler in the matrix, usually at higher filler loading rates [46,65]. Bartoli et al. (2020) also designed a study to investigate the effect of treatment temperature and heating rate of biochar carbonization process on composite properties. They produced biochar using different carbonization temperatures and heating rates. This biochar was incorporated at 2% loading rate into epoxy matrix to study the effect of the pyrolytic thermal history of biochar on composite properties [11]. It was observed that composites incorporated with biochar produced at treatment temperature 400 °C using lower heating rates like 5 and 15 °C per minute had a detrimental effect on the Young’s modulus (YM), and a similar effect was observed for Ultimate Tensile Strength (UTS) [11]. Higher heating rate of 50 °C per minute had a positive effect on the Young’s modulus (YM) and Ultimate Tensile Strength (UTS) of the composite. On the other hand, the elongation of composite dramatically reduced on incorporation of biochar produced at a heating rate of 50 °C. At 600 °C treatment temperature, better results were obtained for biochar produced using lower heating rates. A significant effect of different heating rates on the Young’s modulus and the Ultimate Tensile Strength were not observed for composites filled with biochar carbonized at 800 and 1000 °C, respectively. The elongation of composites was observed to have improved on incorporation of biochar produced using higher treatment temperatures [11]. This improvement properties of composites and the significant differences in the observed results is attributed to the different morphologies of biochar which are obtained using different carbonization temperatures [66]. Based on similar objectives, Q. Zhang, et al. (2020) [67] developed high density polyethylene composites filled with rice husk biochar. The biochar was carbonized at 200, 300, 400, 500, 600, 700, 800 and 900 °C, respectively, to study the effect of carbonization temperature on biochar morphology [67]. A comparison between rice husk filled composites and rice husk biochar filled composites having filler and matrix at one-to-one ratio (50% filler loading) was completed to compare the physical properties of the composites. The tensile properties of the biochar filled composites were observed to be better than both neat HDPE and untreated rice husk (RH) filled composites. The reason for poor tensile properties of rice husk filled composites is the incompatibility between the matrix and the filler, due to the polar nature of rice husk [68]. On carbonization, the polarity of the filler goes down due to the removal of polar functional groups, this phenomenon improves as the temperature of carbonization increases. A mechanical interlocking is created between the molten polymer and the biochar as it enters the pores present on the surface of biochar improving the mechanical properties of the composites [5]. In this study a decline in tensile strength was observed for composites filled with biochar carbonized at temperatures 700, 800 and 900 °C. This decline is attributed to deformation of pores on biochar surface impacting the mechanical interlocking, therefore the tensile properties [67]. The Young’s modulus (YM) for the composites also followed a similar trend, the highest YM value of 1.87 GPa was recorded for biocomposites filled with biochar produced at 500 °C treatment temperature. This is also attributed to the pore structure of biochar which improves the stiffness of composite by mechanical interlocking between the polymer and the filler [67]. The viscoelastic behavior of the composites was studied using dynamic mechanical analysis (DMA). It was observed that the storage modulus of the composites decreased on increase in temperature throughout the experiment. This decrease was due to the increase of thermal movement of thermoplastic matrix molecules in the composite [66,68]. It was reported that the storage modulus of all the composites was higher compared to neat HDPE. The highest modulus value was reported for composites filled with biochar carbonized at 600 °C as a result of mechanical interlocking between the matrix and filler. The creep compliance of the composites was also determined in the study. The creep behavior curve also presents information on the elastic deformation, viscoelastic deformation and viscous deformation of the composites [47]. Improved creep resistance was observed in the composites filled with rice husk and rice husk biochar when compared to HDPE. The creep resistance of biochar filled composites was better than rice husk filled composite [67]. The relaxation modulus values were observed to have improved for the biochar filled composites compared to rice husk filled composites and neat HDPE. The relaxation modulus provides information on the stress relaxation behavior of polymers and composites [69]. All the studies discussed here reported improved properties with the incorporation of biochar carbonized at higher temperatures compared to their lower temperature counterparts. This is associated to better matrix and filler interaction, along with better mechanical interlocking in composites having high temperature biochar, as high temperature helps in removal of functional groups from the biochar surface providing more surface area for polymer–filler interaction.

Filler particle size is another important factor that contributes to composite properties. The shape and size of filler can greatly alter composite properties. It is often observed that the higher the aspect ratio of filler the better the properties of the composite. The effect of variable particle size of biochar on the mechanical properties of nylon composites was studied by Ogunsona, Misra, and Mohanty 2017 [39]. The tensile modulus of the composites showed improvement when composites were filled with milled biochar having particle size <500 µm. The modulus however kept on deteriorating as the particle size kept reducing. The tensile modulus however was observed to be increasing in composite filled with milled biochar compared to crushed biochar, which is believed because of greater interfacial adhesion between the matrix and filler having smaller particle size [70]. However, as the particle size of milled biochar kept decreasing the modulus also kept decreasing. As stated in the literature, a factor contributing to the decline in tensile strength is aspect ratio of filler [39]. A larger aspect ratio is believed to have a synergistic effect on the tensile modulus of the composite. Higher tensile modulus is observed in composites filled with filler having higher aspect ratio compared to composites filled with filler having sheet like or globular structures [71]. Similar trends were observed for tensile strength of composites as well. On addition of crushed biochar, a decline in tensile strength was observed unlike when milled biochar was added and an improvement in the tensile strength of the composite was recorded. A decline in tensile strength was observed as the particle size of biochar kept reducing. Addition of crushed biochar results in mechanical failure of composite samples due to the presence of micropores on the surface of biochar that created weak points throughout the composite [39]. Reduced aspect ratio of particles on reduction of particle size results in increased failure due to elastic deformation in the direction of force [58]. Flexural properties of the composite also showed similar results. However, the overall flexural properties of the composites were better than neat PA 6, 10. The composites filled with milled biochar having a particle size <500 µm showed the best properties, a further decline in properties was observed as the particle size kept decreasing [39]. The impact properties on the other hand showed a different trend and it was observed that on addition of milled biochar the impact properties improved, and it kept improving as the particle size reduced progressively. A reason attributed to this is the reduction in ductility of the composite and hampered plastic deformation in the composite [39], reduction in particle size promotes shape homogeneity and reduction in defects also improves the impact properties of the composite. The incorporation of more globular particles results in higher energy dissipation and higher impact strength owing to the shape of the filler [72]. In a similar experiment, H. Zhang et al. (2018) [7] reported the results for mechanical properties of polypropylene (PP)/bamboo particles (BP)/ultrafine bamboo biochar (UFBC). With the increase in content of UFBC from 15% to 30% there was a considerable improvement in the tensile strength and elongation at break values of the composite. This enhancement is assumed to be caused by the intrinsically strong biochar that is adding to the tensile strength of the composites by synergistic interaction between the three components. The elongation at break also experienced a similar trend. The composites were prepared with two different particle sizes of biochar P1 and P2. The composites filled with P2 showed better properties compared to composites filled with P1. The flexural strength of the composites showed an improvement at a loading of 20% for biochar particle size P1 and no difference was observed for filler loading lower or higher than 20%. Composites filled with biochar particles P2 showed a steep increase in flexural properties with increase in biochar loading. The moisture resistance of the composites was also evaluated. The moisture resistance of the composites seemed to be increasing at 5% biochar loading but then with increasing biochar loading a decline in the moisture resistance was observed. In case of particle size, no clear trend in the property was observed. As noted in the studies, the higher aspect ratio of filler proved beneficial for certain mechanical properties like tensile strength and modulus. On the other hand, smaller (globular) particle size of filler was shown to have improved the impact properties. Through these results it can be understood that based on the desired property the size and shape of the filler can be altered, in turn customizing the overall composite properties.

A few studies with some unique aspects unlike the ones mentioned above are summarized here. Ferreira et al. (2019) [41] designed a unique study and introduced chemical treatment of biochar prior to addition as a filler. In the study they characterized the three different composites filled with carbon black, bagasse biochar milled for 72 h (SBB-72 h) and milled and treated biochar (rSBB-ABL-72h) at 1% and 5 wt% loading rates. The study reported close values for Young’s modulus for all the composites, however, the values for composites filled with only milled biochar was lowest among the three. A better adhesion between the rSBB-ABL-72 h biochar and the matrix is attributed to the lack of oxygenated carbon groups [41]. The authors stated that factors like polarity difference between matrix and filler, particle size and morphology of additives have a predominant effect on the mechanical properties of the composites [37,71,72]. It was observed that the mechanical properties of composites filled with rSBB-ABL-72 h biochar were relatively more similar to the values obtained for carbon black filled polyethylene composites, compared to SBB-72 h biochar filled composites [41]. Similarly, Li et al. (2016) [36] developed UHMWPE composites filled with biochar with segregated filler network where the filler network is confined in a certain space and is not dispersed throughout the composite volume making it a very unique study design. An increase in the tensile modulus of the composites was observed with increase in filler loading rate. At the highest loading rate of 9 wt% a Young’s modulus value of 388.4 MPa was recorded which is 18.9% higher than neat polymer [36]. The tensile strength however showed improvement on addition of filler, at 3% filler loading, an increase of 10% was observed compared to neat UHMWPE, but it kept deteriorating with increasing loading rate. The improvement in tensile properties of the composite is due to the good interfacial adhesion between the charcoal filler and UHMWPE polymer matrix [36]. The segregated networks of filler have added stiffness to the composite by restricting the movement of polymer chains in the composite [73,74]. The occurrence of voids along the segregated pathways on increase of filler content was observed, leading to the impairment of mechanical properties [36]. A decrease in ductility was observed in composites, compared to neat polymer matrix. Another one of a kind study was designed by Behazin et al. (2018) [4] to study the mechanical properties of biocarbon filled composites heat aged for 1000 h. The mechanical testing results were compared to the specified values set by automobile manufacturers, the values were set precisely ±15% of the set values. The mechanical properties of the composites remained the same through the periods of heat aging except for the control sample which showed about 85% failure at 500 h of aging [4]. Behazin et al. (2018) [4] reported no loss in tensile properties of heat stabilized samples throughout the heat aging period and reported around 5% improvement in yield strength compared to the lab conditioned samples. This improvement is attributed to increase in the β crystals content. Compared to the laboratory conditioned samples the heat aged samples irrespective of the presence of stabilizers showed reduced impact properties. This study is unique since heat aging of composites is an important aspect that needs to be assessed for long term application of polymers and composites.

Arrigo, Bartoli, and Malucelli (2020) [46] reported enhancement of the tensile modulus for both the composites fabricated using melt mixing and solvent casting. The increase is attributed to the uniform dispersion of filler thoughout the matrix [46]. A decline in tensile strength of the composites was reported, premature failing of the composites due to presence of voids was stated as the reason by the authors [46]. The uniqueness of the study is the comparison of two different fabrication methods and the effect of these methods in the composite properties.

The mechanical properties as reported in the studies addressed here are summarized in Table 2.

Overall improvement in composite mechanical properties was observed on incorporation of biochar into the composite system. A very important contributor to enhanced mechanical properties is the mechanical interlocking between the biochar filler and polymer matrix. The presence of pores on the biochar surface allows the molten polymer to enter into the pores forming a crosslinked structure that creates a strong connection between the matrix and the filler. The studies discussed above mentioned that even in the presence of compatibilizer this mechanical interlocking was an important reason for enhancement of mechanical properties of biochar filled composites [5,28,39,66,75,76]. Compatibilizer was shown to be helpful in improving composite properties, but no interaction between the biochar and compatibilizer could take place due to the lack of functional groups in biochar [26,39,77]. Biochars produced at higher temperatures have shown better interfacial adhesion compared to biochars carbonized at lower temperatures due to the abundance of pores on biochar surface attributed to the better quality of biochar filled composites. A considerable reduction in the ductility of the composite samples is observed as addition of biochar makes composites stiffer than the neat polymer. This property is further enhanced by addition of natural additives like wood fiber or flour. Particle size of biochar has not been selected as a crucial property but has been reported to have an impact on the final properties of the composites [28,78]. The DMA also provided an overview of the thermomechanical properties of the composite. It provides an opportunity to look at the rheology and mechanical properties of the composite with respect to temperature. An improvement in viscoelastic properties was also observed on incorporation of biochar [67]. The DMA results are discussed in detail in the next Section 3.2.

### 3.2. Thermal Properties

Addition of biochar to polymers is expected to positively impact the thermal properties of polymer matrix. The thermal stability, melting point, crystalline properties and flammability the composites are analyzed using methods like thermogravimetric analysis (TGA), differential scanning calorimetry (DSC), etc.

**Figure 6:**
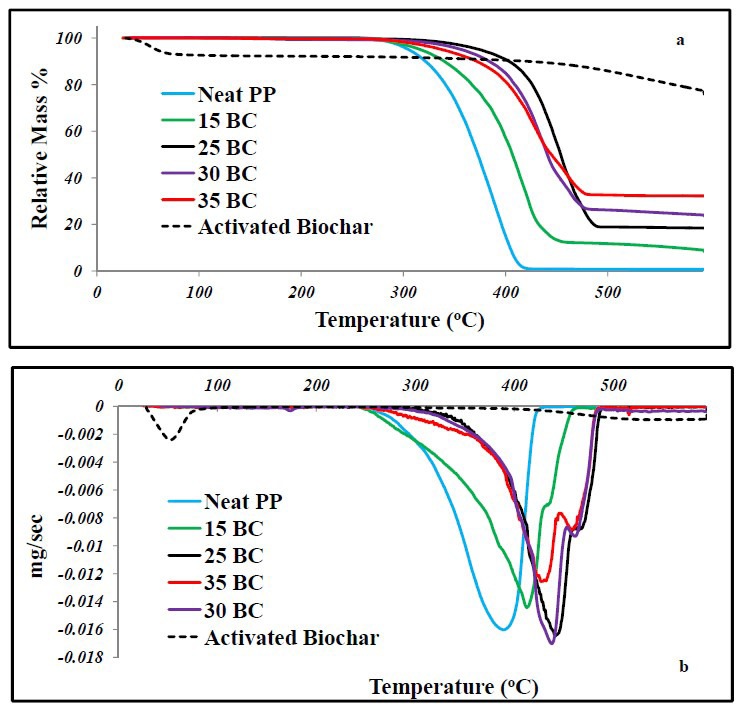
The TGA and DTG curves of biochar filled polypropylene composites, (**a**) TGA (mass loss) curve and (**b**) DTG curve. A significant shift in the degradation temperature of the composite in comparison to neat polymer can be observed. The image is modified from [5] with permission from Elsevier publications 2016 for reusing and modifying the image.

Thermal degradation studies completed for the biochar filled composites in different studies indicated increased thermal stability on addition of biochar, compared to thermal degradation of neat polymer. A detailed discussion of the TGA results is as follows. The TGA results reported by Das, Bhattacharyya et al. (2016) [5] indicated that the composites with biochar had higher thermal stability compared to neat polypropylene. The temperature of degradation increased with the addition of biochar in the composite indicating improved thermal properties. The onset of thermal degradation for neat PP was observed at ~390 °C, however, with the addition of biochar the temperature for degradation increased to ~412 °C just at 15% biochar content [5]. The trend showed an increase with increase in biochar content along with increased char or residue. The TGA curves are presented in Figure 5. It can be observed in Figure 6a that the initiation of thermal degradation of composites filled with 30% and 35 wt%, biochar occurs at a lower temperature compared to 25 wt% biochar filled composites. The composites, irrespective of filler concentration have a higher temperature of degradation compared to pure polymer, however, the trend of onset of thermal degradation is not in concordance with the order of filler concentration. The residue retention is following a linear trend, that is, with higher biochar loading the residue retention is higher, but the same trend is not observed for the onset of degradation temperature as 35% biochar filled samples have an onset of thermal degradation temperature lower than 25% biochar filled composites. This phenomenon is very interesting, however, a clear reasoning for the occurrence of this is not explained in the literature and it would be interesting to learn more about this anomaly in the composite system

Das, Bhattacharyya, and Sarmah (2016c) [14] reported the thermal properties of composites composed of 24% biochar, wood and MAPP (0–3%). The TGA thermograms showed two peaks of weight loss due to degradation, one at ~370 °C and one at ~450 °C, respectively. The peak at ~370 °C is due to the degradation of cellulose in wood, while the peak at ~410–~450 °C was due to the degradation of PP [79]. In this case, the decomposition of composites was inversely related to the amount of MAPP present, that is, the composite with 3% MAPP had the lowest degradation temperature. This happens due to the better interfacial adhesion between wood and PP due to the presence of MAPP, resulting in better heat dissipation, and the higher the amount of MAPP the better the interfacial bonding [42]. This indicates the presence of a higher compatibilizer amount is not necessarily beneficial towards composite thermal properties. Similarly, Das, Bhattacharyya, and Sarmah (2016) [28] reported TGA analysis of neat PP and hybrid composites filled with wood and biochar. The TGA data showed onset of decomposition of neat PP before 300 °C and it leaves no residue post decomposition [28]. Due to the presence of thermally stable biochar, the composites left a higher amount of residue after decomposition. It was observed that the composites having no wood in them were relatively much more thermally stable when compared to composites having wood as a component. The derivative curves for the composites showed similar trends. The composites without wood were more thermally stable. The composites with wood had two decomposition peaks, the first peak being for decomposition of cellulose in wood at a much lower temperature like 370 °C [28]. This peak was not observed for the composites that did not have wood as one of the composite components. However, the overall thermal stability of the composites was enhanced on addition of biochar. Li et al. (2016) [36] analyzed the nano-bamboo charcoal filled UHMWPE composites with segregated networks for thermal stability using TGA. A shift in thermal degradation temperatures to a higher temperature was observed for the charcoal filled composites indicating improved thermal properties. No residue was left post the thermal degradation of neat UHMWPE, but an increase in the residue retention was observed for the charcoal filled composites indicating improved thermal stability. Similar results were obtained for thermal properties in studies done by Meng et al. (2013) [80]. In the study, the effect of bamboo charcoal powder on the curing characteristics, mechanical and thermal properties of styrene butadiene rubber was studied. The results indicated improved thermal stability of the composite with biochar incorporation [80]. Similarly, Abdul Khalil et al. (2010) [81] in their study incorporated carbon black derived from natural sources like bamboo, coconut shell and empty palm fruit bunch into epoxy matrix to evaluate its effect on mechanical and thermal properties. They also reported improved thermal stability for all the composites filled with the different biochars [81]. Q. Zhang, Zhang, Lu, et al. (2020) [82] developed rice husk biochar filled composites. The TGA analysis of neat HDPE was observed to have a thermal degradation temperature of 480 °C [83,84,85]. The DTG curve of the composites filled with biochar carbonized at 200, 300 and 400 °C showed peaks mainly due to pyrolysis and volatilization of cellulose and hemicellulose at 330 and 380 °C, respectively, suggesting the incomplete carbonization of feedstock with the respective temperatures [67]. The advent of thermal degradation of composites took place at a higher temperature of 490 °C, showing improvement in thermal properties of the composites compared to neat HDPE. A higher residue was also retained for biochar filled composites, filled with filler carbonized at temperatures 600, 700, 800 and 900 °C, respectively. This phenomenon indicates increase in the thermal stability of polymer on addition of filler [83,86]. Arrigo, Bartoli, and Malucelli (2020) [46] reported the TGA of biochar filled polylactic acid (PLA) composites. The TGA was performed by heating the samples to 600 °C in nitrogen atmosphere. A weight loss step was observed for solvent cast composites which is believed to be the loss of residual solvents forming the films [86]. A decrease in degradation temperature was observed for both the composites at a higher filler loading rate. This detrimental effect of addition of biochar on the thermal properties of PLA has also been reported in the studies Ho et al. (2015) [73] and Moustafa et al. (2017) [87] and the cause is believed to be potassium present in biochar which affects the thermal decomposition of polymer matrix [74,88]. An increasing phosphorous content can further deteriorate the thermal stability of PLA polymer [46]. It is also stated by the authors that the residual hydroxyl groups on biochar can lead to hydrolytic and back biting reactions mechanisms in PLA [89,90]. Ferreira et al. (2019) [41] studied the effect of the three different fillers carbon black, SBB-72 h biochar and rSBB-ALB-72 h biochar on the thermal stability of polyethylene. An improvement in the thermal stability was observed for the composites when compared to neat polyethylene. It is stated that the improvement in thermal properties of biochar filled composites is 15% higher than the values reported for commercial carbon black filled composites [41]. A similar effect of addition of carbon black nano-particles was observed on polypropylene and the enhancement in thermal stability was attributed to changes in the degradation mechanism and kinetics of the polymer on addition of filler [91,92]. All the studies discussed here have reported a shift in the thermal degradation temperature to a higher temperature along with increase in the residue retention. These results are consistent in almost all the studies and are indicative of the positive impact of biochar on enhancing the thermal stability of the composites.

**Figure 7:**
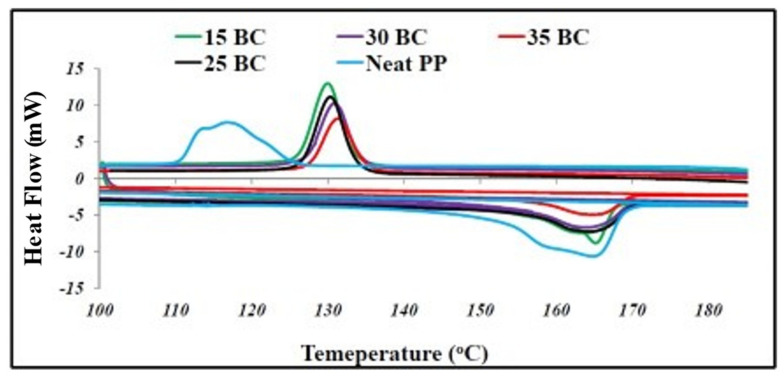
DSC thermogram of biochar filled polypropylene composites, no change in melting temperature was observed but a rise in crystallization temperature was observed due to the nucleation effect of biochar. Figure taken and modified from [5] with permission from Elsevier publication 2016.

The effects of incorporation of biochar filler on the melt characteristics and crystallinity of the polymer are analyzed using differential scanning calorimetry (DSC). The results of different studies are summarized here. Das, Bhattacharyya et al. (2016) [5] reported that the DSC thermograms show no change in the melting temperature of polypropylene, but there was an increase in the energy required to melt the biochar filled biocomposites. On the other hand, there was an increase in the crystallization temperature that is believed to be the effect of the biochar particles acting as nucleating agents and resulting in crystal growth. Another phenomenon observed in the biochar filled biocomposites was that the intensity of crystallization peak reduced with the increase in filler quantity. This resulted in less energy required for crystallization. The DSC thermogram of the polypropylene composites is presented in Figure 7.

The DSC thermograms of the pine biochar filled polypropylene composites reported by Ikram et al. (2016) [28] showed no change in the melting temperature as well in comparison to neat polypropylene. The energy needed for melting, however, increased on inclusion of thermally stable biochar in the composites. A change in crystallization temperature was observed as it moved to a higher temperature due to the nucleation effect of biochar resulting in crystal growth. The results are similar to the results obtained by Das, Bhattacharyya et al. (2016) [5]. H. Zhang et al. (2018) [7] evaluated the thermal properties of polypropylene/bamboo particles/ultrafine bamboo biochar. On addition of biochar specifically biochar P2 there was an increase in crystallization temperature. This is attributed to the nucleating effect of biochar. An improvement in melting temperature was also reported indicating enhanced thermal stability. The crystallinity of the composites also improved. Nylon composites filled with clay filler also showed similar results when analyzed using FTIR and XRD analysis [92]. It was observed that addition of clay filler enhanced formation of γ phase crystals which is a relatively less ordered crystal structure causing reduction in crystallinity of the polymer [40,93]. The reason behind the favored phase formation is still not understood, however, probable reasons behind this phenomenon suggest that conformational changes in the structure are caused by fillers by forcing the amide groups of nylon onto out of plane formation resulting in reduction of hydrogen bonded sheets in the polymer [94,95]. Behazin et al. (2018) [4] reported the crystalline phase alterations in control and heat stabilized heat aged composite samples. The DSC thermograms show and increase in the crystallinity of control samples that flattened after 500 h of aging. The increase in the crystallinity of the control samples is attributed to thermo-oxidative chain scission and annealing. Ferreira et al. (2019) [41] studied the effect of the three different fillers carbon black, SBB-72 h biochar and rSBB-ALB-72 h biochar on the thermal stability of polyethylene. An improvement in the thermal stability was observed for the composites when compared to neat polyethylene. It is stated that the improvement in thermal properties of biochar filled composites is 15% higher than the values reported for commercial carbon black filled composites [41]. A similar effect of addition of carbon black nano-particles was observed on polypropylene and the enhancement in thermal stability was attributed to changes on the degradation mechanism and kinetics of the polymer on addition of filler [91,92]. The DSC analysis of the composites reported no major impact of fillers on the transition temperatures of the polymer. The enthalpy values for fusion and crystallization, however, were observed to have decreased [41]. This change in enthalpy in the polymer is due to the heterogenous nucleation effect onset by the addition of fillers to the polymer [96,97,98]. Even in this scenario the composites filled with treated biochar rSBB-ABL-72 h produced results similar to the results obtained for composites filled with commercial carbon black, indicating its ability as a potential carbon black replacement [41]. Q. Zhang, Zhang, Lu et al. (2020) [67] developed high density polyethylene (HDPE) composites filled with rice husk and rice husk biochar. Neat HDPE shows endothermic melting and exothermic crystallization characteristics [98]. On addition of the fillers rice husk and rice husk biochar to the matrix, a shift in the melting and crystallization behavior of the polymer was observed. It was observed that on introduction of filler the melting temperature experienced a decline, while the crystallization temperature was observed to have increased [67]. The shift in the crystallization temperature is explained by the nucleation effect of filler on the polymer promoting crystal growth and facilitating early crystallization of HDPE [99]. Arrigo, Bartoli, and Malucelli (2020) [46] DSC results stated that in the melt mixing composites a decrease in the glass transition temperature (Tg) and cold crystallization temperature (Tcc) was observed, owing to the increased mobility of PLA molecules as a result of degradation during processing [46]. The percentage crystallinity of the composite remains unchanged and presents an amorphous structure [46]. The solvent casting composites showed lower Tg, Tcc and melting temperature Tm indicating enhanced crystallinity, which was presented in composites with filler loading of 1% and 2.5% [46]. At higher filler loadings, such a dramatic crystallinity was not observed as a higher concentration of filler interferes with the process of crystallization [46]. The DSC results of the studies indicate the change in crystallization parameters in the biochar filled composites. All the studies here have reported the onset of nucleation effect of biochar on the polymer enhancing the crystallization temperature and/or the energy required for crystallization. In some studies, the increase in melting temperature has been reported and in some a decrease in melting and crystallization enthalpies have been reported. All in all, the addition of biochar to the polymer composites has indicated improved thermal properties. Flammability of composites is an important parameter as the range of flammability can be taken into consideration when determining the application of polymers. In most cases, lower flammability is good for application where there are chances of fire hazards for instance. The flammability of composites was studied by Das, Bhattacharyya et al. (2016) [5], to see if biochar acts as a flame retardant and has the potential to replace conventional chemical flame retardants used with polypropylene. The composites were tested for flammability and data for time to ignition (TTI), peak heat release rate (PHRR) and total heat release rate (THR) was obtained. It was observed that the PHHR was significantly reduced on addition of biochar. The TTI also reduced with increased biochar content. The THR values did not show much change. The CO and CO_2_ production was significantly reduced. Addition of biochar and wood reduced the PHHR value in composites, this was not in conjunction with the amount of MAPP in the sample. It is believed that the C–C covalent bonds present in biochar [100] result in the thermal stability preventing transfer of heat to the polypropylene matrix. This phenomenon is evidence for the use of biochar as a fire retardant additive and can replace conventional fire retardants which compromise the mechanical properties of the composite [42]. Das, Bhattacharyya, and Sarmah (2016) [42] reported significantly lower production of CO_2_ and CO, which is attributed to the presence of thermally stable biochar in the composites. On addition of wood and biochar the PHRR value of neat PP is reduced by almost 50% due to the formation of a layer of char on the surface of the polymer hindering heat transfer [28]. The composites not containing wood had slightly higher values for PHRR and THR compared to those containing wood, as presence of lignin in wood facilitates char formation which along with thermally stable biochar does a great job in hindering heat transfer enhancing thermal stability. Compared to neat PP the TTI of composites containing wood reduced due to earlier onset of thermal decomposition of wood compared to polypropylene and biochar [28]. The flammability results also indicated improved thermal stability of biochar filled polymer composites.

The effect of biochar on the viscoelastic properties of the polymer and the composites can be studied using dynamic mechanical analysis (DMA). These properties essentially indicate the effect of filler addition on the viscosity along with the elastic (properties inherent to the polymer matrix) properties of the composite. The viscoelastic properties of polypropylene composites filled with biochar were analyzed by Behazin et al. (2017) [38] using dynamic mechanical analysis (DMA). Two glass transition peaks were observed for polypropylene and POE indicating the two polymers being thermodynamically immiscible [38]. Addition of biochar led to shifting of the peak towards lower temperature, this was more proficient in composites having LtBioC, and was not observed for polypropylene. This is believed to be due to the free movement of POE chains around LtBioC, due to weaker interaction between the two. The dynamic mechanical analysis (DMA) of the composites showed a shift in glass transition temperature Tg of the polymer to a higher temperature on incorporation of biochar. The Tg value kept increasing with the decrease in biochar particle size. The heat deflection temperature (HDT) was seen to increase on addition of biochar filler in the matrix and it kept increasing as the particle size of biochar was reduced. This phenomenon is explained to occur due to the reduction of interparticle distance limiting the radius of gyration of polymer, hence making it hard to displace [39]. The DMA results are also in conjunction with the other thermal properties results and indicate improvement in composite properties with incorporation of biochar filler.

A shift in the thermal degradation temperature of composites when compared to neat polymer was observed in most of the studies through the TGA analysis. Through DSC it was observed that biochar acts as a nucleating agent in the matrix leading to heterogenous crystallization of the matrix and an increase in the crystallization temperature of the polymer. The flammability of the polymer was reduced on incorporation of the biochar which will pave the way for enhanced applicability of the polymer. A positive impact on thermal properties was observed on incorporation of biochar filler. Improved thermal stability of the composite is helpful in increasing the applicability of the polymer composites which was otherwise restricted due to the thermal properties of the polymer on its own.

### 3.3. Electrical Conductivity in Composites

**Figure 8:**
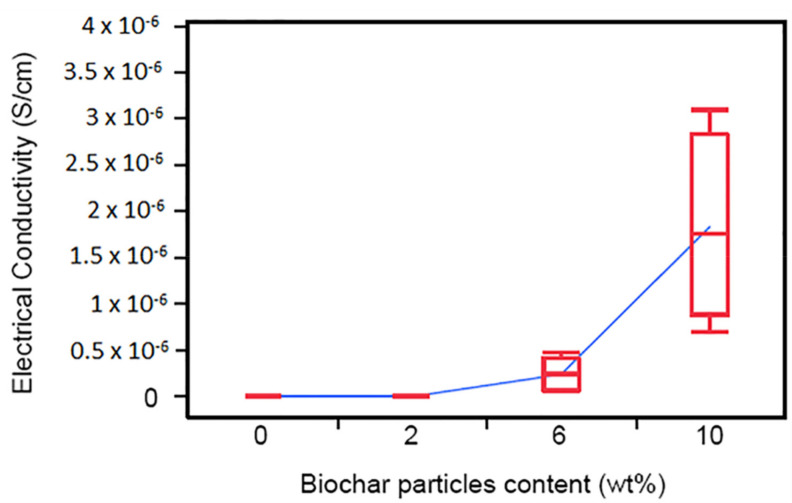
Electrical conductivity of biochar filled PVA composites filled with 2%, 6% and 10% biochar filler, respectively. An increase in conductivity is observed with increase in loading rate of biochar. Figure taken from [27] with permission to use from SagePub Publications 2016.

Electrically conductive polymer composites (ECPC) are developed by incorporation of conductive filler material into a non-conductive matrix [101,102,103]. Electrically conductive polymer composites are manufactured using carbon-based fillers like carbon nanotubes, carbon fibers, carbon black, etc. Manufacture of these synthetic fillers is quite time and energy intensive. Biochar when carbonized at high treatment temperatures above 500 °C shows electrical conductivity. Gabhi, Kirk, and Jia (2017) [104], R. Gabhi et al. (2020) [105] in their studies observed that carbonization of wood blocks at higher temperatures of 1000 °C leads to a subsequent increase in carbon content in the char. This temperature of pyrolysis and carbon content plays an important role in electrical conductivity of biochar. The study reported that biochar monoliths carbonized at 1000 °C had electrical conductivity between the range of 2300–3300 S/m [105]. Electrically conductive composites developed by Nan et al. (2016) [33] reported electrical conductivity in biochar filled composites fabricated using solution casting method. As per their study, at a filler concentration of 6 wt%., transition from non-conductive to electrically conductive was observed and at 10 wt%. filler concentration a conductivity of 1.833 × 10^−3^ S/cm [33]. The electrical conductivity value for composites filled with 10% biochar were comparable to electrical conductivity values obtained for composites filled with 1% single walled carbon nanotubes and 6% graphene [33]. The increasing trend in electrical conductivity with increase in biochar concentration in the composites is presented in Figure 8.

Similarly, Li et al. (2016) [36] have reported a typical percolation behavior for their composites by drastic increase in conductivity at filler concentrations of 0.8 and 2.3 vol%. The percolation threshold is the quantity of filler that is needed to make a non-conductive matrix conductive by formation of conducting networks in the composite system [106]. The percolation threshold for conductivity was determined to be 2.0 vol% corresponding to 2.6 wt% of filler, the value was reported to be higher than percolation threshold values reported for carbon nanotube, graphene nanosheets and carbon black filled composites developed using the same matrix and composite preparation method [107,108]. The high percolation value is believed to be due to lower inherent conductivity, high particle size and low aspect ratio of filler [36]. Electrical conductivity value of 1.1 × 10^−2^ S/cm was recorded at 7% charcoal loading composite. Gao et al. (2008) [109] reported a conductivity value of 2.0 × 10^−2^ S/cm at a percolation threshold of 0.88 vol% for CNT filled UHMWPE composites fabricated using an alcohol assisted dispersion method combined with hot compression method. Yan et al. (2014) [110] were able to produce a conductivity value of 3.4 × 10^−2^ S/cm at a filler concentration of 0.66 vol% in reduced graphene filled UHMWPE composites. A higher concentration of filler is required to achieve such high electrical conductivity values in case of charcoal/biochar fillers when compared to the studies using synthetic carbon-based fillers. The authors have suggested that even if the loading rate of filler is high in the study the developed composite has a lot of advantages like being sustainable, cheap, less time consuming and has a potential for commercial application [36]. In an interesting study, Khan et al. (2017) [34] reported an increase in the real part of dielectric constant and electric conductivity on increasing filler concentration. A higher loading rate of 10% was needed to observe an enhancement in conductivity of composites, while for MWCNTS the change could be observed even at low concentrations of the filler [111,112,113,114,115]. The high aspect ratio of MWCNTs was also attributed to the high electrical conductivity compared to the three dimensional structure of biochar [34]. It was observed that the removal of several functional groups from the surface of biochar also contribute to the lower permittivity and conductivity of biochar. Biochar 20 wt% and MWCNTs 4 wt% were showed to have similar values for the microwave properties, but with a very high difference in loading rate [34]. The more graphitic structure of MWCNTs compared to the structure of biochar could be one of the reasons behind this difference in property as per the authors [34]. In the study designed by Li et al. (2018) [36] they developed biochar filled polyethylene composites using biochar carbonized at 1100 °C. The composites were developed with of 60, 70 and 80 wt% biochar loading rates. The study reported excellent conductivity value of 107.6 S/m at 80 wt% biochar loading rate. This is by far the highest conductivity value obtained for biochar filled polymer composites [45]. Like other studies, Giorcelli and Bartoli (2019) [44] developed biochar from coffee waste by carbonizing the biomass at 400, 600, 800 and 1000 °C temperatures. The biochar was incorporated in epoxy matrix to form composites at different loading rates of 5, 10, 15 and 20 wt%. At 20 wt%loading rate the electrical conductivity value for composites filled with biochar carbonized at 1000 °C was reported to be 2.02 S/m which was four orders more than the electrical conductivity value reported for 20 wt% carbon black loading [44]. The electrical conductivity values are summarized in Table 3.

It was observed that biochars pyrolyzed at high carbonization temperatures have a significant impact on conductivity of composites and have done a great job in making non-conductive polymer electrically conductive as indicated in the studies. It is observed that the conductivity follows a positive trend and shows increase on increase of loading rate of filler. Enhanced mechanical properties were reported in all the three studies on incorporation of biochar in the polymer matrix. The review for applications of biochar as an electrically conductive filler indicated that the research is still in its very initial stages. The number of articles reporting the application of biochar as a fortifying filler is much higher compared to papers reporting the use of biochar as an electrically conductive filler. There are several other factors like biochar composition, morphology, source of feedstock, etc., that could have an effect on the electrical conductivity and are worth exploring. There is immense scope in this research and with time there is definitely going to be more focus on application of biochar as an electrically conductive filler.

### 3.4. Morphological Properties

The study of morphology of the composites gives us information on how good the interfacial interaction between the filler and the matrix is. The morphological properties are mainly studied using scanning electron microscopy. The fractured surface is subjected to viewing under the microscope which gives us information about the filler distribution and interaction with the matrix.

Lignocellulosic biomass on carbonization results in formation of a honeycomb like structures with pores. When this biochar is incorporated into a polymer matrix the molten polymer enters these pores establishing a mechanical interlocking that improves the mechanical properties of the composites [5,42,83,116].

**Figure 9:**
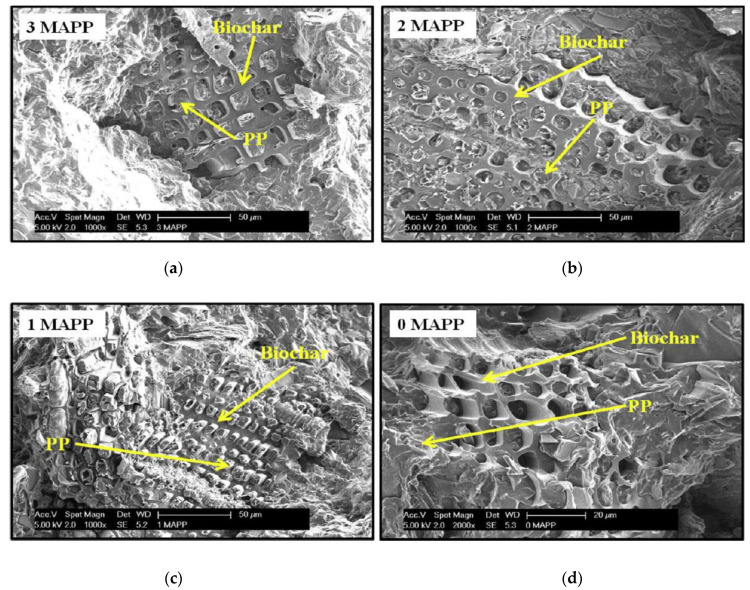
The SEM images of fractured surfaces of biochar filled polypropylene composites (**a**) 3 MAPP (**b**) 2 MAPP (**c**) 1 MAPP (**d**) 0 MAPP, the images show mechanical interlocking between the filler and the matrix polymer even at 1% concentration of compatibilizer attributed to its excellent mechanical properties. Figure taken from [42] with permission to use from Elsevier Publications 2016.

As described in earlier sections, compatibilizers like MAPP have shown synergistic effects on the mechanical properties of composites. The effect of MAPP on the composite mechanical properties can be presented better on a morphological level. The studies discussed in this section explain how the presence of MAPP affected the final properties of their respective composites. Das, Bhattacharyya et al. (2016) [5] reported uniform distribution of biochar filler in the PP matrix in 15% biochar filled samples. This is attributed to the physical mixing of samples prior to extrusion. Infiltration of biochar pores with molten PP was observed in the SEM images, resulting in interlocking between the polymer matrix and the filler [5]. This is believed to be the reason behind improved mechanical properties on addition of biochar filler to polypropylene matrix. The SEM analysis results for PP, biochar, wood and MAPP composites showed mechanical interlocking between biochar and polypropylene as the molten polymer has infiltrated the pores present on the biochar surface. Due to the absence of functional groups on the surface of biochar, MAPP assisted chemical bonding of biochar and PP could not be formed. In the presence of 3% and 2% MAPP there is a good interfacial adhesion between polypropylene and wood, while in the case of 1% MAPP interfacial bonding between PP and wood was poor. It is suggested that addition of biochar can compensate for the poor interfacial adhesion [42]. The SEM image in Figure 9 shows the mechanical interlocking between the biochar and the polymer matrix.

Similarly, the SEM images of the composites developed by Ikram et al. (2016) [28] showed that the composites having wood and compatibilizer MAPP showed good bonding between the wood and polypropylene matrix whereas the composites having wood and no MAPP showed debonding. Mechanical interlocking between the molten polymer and biochar due to the abundance of pores on the biochar surface resulted in enhanced mechanical properties [5]. Behazin et al. (2017a) [37] studied the correlation between morphology of the composites and its properties. The evaluation was carried out keeping the compatibilizer concentration constant at 7.5%. When no compatibilizer or MAPE was used as a compatibilizer, voids were observed around the filler particles; this was not observed when MAPP was used [37]. In the absence of compatibilizer or in presence of MAPE, the rubbery phase of the matrix POE encapsulated the filler particles resulting in the voids, which did not happen in the presence of MAPP. A closer look at the morphology of the composites showed that when MAPE is used, it changes the shape of POE from semi-spherical to elongated fibrillar shape [37]. This change in morphology is considered a reason for improvement of impact properties on addition of MAPE.

Temperature of carbonization of biomass is a very important factor impacting the structure of the final product. A higher temperature of carbonization results in a biochar with greater surface areas and a more prevalent pore structure that when incorporated into a composite has synergistic effects on the overall composite properties. Li et al. (2016) [36] reported the SEM images for nano-bamboo charcoal. The average particle size for the filler was reported to be 606 nm [36]. The charcoal particles showed irregular shape with a porous structure including narrow micropores with width less than 2 nm and wide macropores with width greater than 50 nm [117]. The SEM images of fractured surface of the composites showed the dispersion of charcoal filler on the interface owing to the fabrication process that caused this particular segregated distribution pattern. It is concluded that segregated networks of filler can be obtained for natural filler using the fabrication method discussed in this study [36]. Behazin et al. (2017) [38,118] reported the SEM analysis of fractured surface of the composites; the SEM images showed good distribution of filler in the matrix in case of 20% HtBioC filled composites. In the case of LtBioC, the images indicated poor compatibility between matrix and filler. Ogunsona, Misra, and Mohanty (2017a) [40] developed biochar filled nylon 6 composites to study the impact of interfacial adhesion on the composite properties. The atomic force microscopy (AFM) analysis of the composites provided the DMT modulus for the biochar embedded in the matrix. It was observed that the modulus values of the biochars were directly corresponding to the temperature of pyrolysis [100]. The DMT modulus value for B1 was reported to be in the range of approximately 4–5 GPa, the fluctuations of the modulus value are related to the turbostatic structure of biochar which is composed of both crystalline and amorphous regions. The peak intensity is increased when the probe comes across a highly ordered crystalline region in the biochar and vice-versa when a not very ordered amorphous region is encountered [40]. Ogunsona, Misra, and Mohanty (2017a) [40] reported a high fluctuation in the peaks of B1 due to the presence of numerous small crystalline phases distributed across the biochar system. The DMT modulus of B2 was reported to be approximately 13–19 GPa and in the analysis fewer and pristine peaks were observed due to the presence of larger and well developed crystalline phases in B2 owing to the higher temperature of pyrolysis [40,119,120]. The SEM analysis of biochar B1 and B2 filler showed the particle size was under 5 µm even for agglomerates, particle size as low as nanometers was also observed. The presence of particles in nanometric level is attributed to the reinforcement ability of the biochars. Formation of agglomerates is also reported and this is attributed to presence of Van der Walls attractive forces causing agglomerate formation [121]. The formation of agglomerates is higher in B1 as compared to B2 owing to its lower temperature of pyrolysis causing presence of residual functional groups on the surface of biochar. The reduction of biochar particle size to nanometric levels is attributed to the milling conditions that can lead to increase in pressure exerted on the particles along with increase in temperature exacerbating the breakdown of biochar particles while milling [122]. The SEM analysis of fractured surfaces of the composites show better wetting in the case of B1 filled biochar composites as the polymer was seen to form a layer around the filler indicating good interfacial adhesion. In the case of B2 filled composites, the SEM images showed presence of voids indicative of poor interfacial adhesion between polymer and filler. The presence of functional groups on the surface of B1 biochar is believed to have facilitated good interfacial adhesion by interacting with the functional groups present in nylon matrix [123]. The surface properties of biochar which are a result of temperature of pyrolysis have played important role in determining the interfacial adhesion between the polymer matrix and filler affecting the properties of the composites. The SEM analysis of the biochar and biochar (HT) filler filled epoxy composites developed by Giorcelli et al. (2019) [64] showed uniform dispersion of filler in the matrix at lower loading rates. Agglomeration of filler can be observed in composites filled with higher loading rates of biochar filler. It was observed that the porous morphology of biochar was lost during the milling of biochar into a fine powder. It was observed that the filler particles were embedded very well in the matrix and this facilitated the improvement in mechanical properties of the composites. The microstructure of fractured surfaces of rice husk filled and rice husk biochar filled composites was observed under scanning electron microscopy (SEM) using different magnifications to study the interfacial morphology and the filler–matrix interactions by Q. Zhang, Zhang, Lu, et al. (2020) [67] who reported that biochars carbonized at lower temperatures of 200 and 300 °C showed a similar morphology to that of rice husk filled composite. The microstructure of these composites showed the filler wrapped in HDPE matrix [67,124,125]. The SEM images of composites filled with biochar carbonized at 400 °C showed presence of both rice husk and rice husk biochar, indicating incomplete carbonization of feedstock. The composites with biochar carbonized at 500 and 600 °C showed mechanical interlocking between the matrix and the polymer; this is reflected in the mechanical properties discussed in earlier sections, as composites having biochar produced at 500 and 600 °C showed the best tensile and viscoelastic properties [67]. Composites filled with biochar produced at 700, 800 and 900 °C showed the destruction of the micropore structure on the surface of biochar hampering the mechanical interlocking [67]. This resulted in the decline in mechanical properties of the composites filled with biochar produced beyond 600 °C. The microstructure of composites filled with the biochar produced at higher temperatures like 800 °C shows a lot of cracks not supporting the formation of the physical interlocking of the molten polymer matrix and filler [67]. Arrigo, Bartoli, and Malucelli (2020) [46] studied the microstructure of their composites using SEM. A homogenous distribution of filler was observed for both the processes, a reduction in filler size was observed in composites fabricated using melt mixing [46]. The intense shear forces applied on the samples during melt mixing fabrication process resulted in the reduction of particle size of filler [126].

The morphological studies showed the structure of biochar and its interaction with the polymer matrix in detail. As it can be seen in most of the studies, the improved properties can be attributed to the mechanical interlocking between the biochar filler and polymer matrix. Factors like high temperature facilitate the formation of this well-formed honeycomb structure facilitating the improvements on incorporation of biochar. A similar effect is observed in increasing biochar concentration in matrix, usually up to a certain extent until an optimal amount is reached. The morphological studies give us an in depth overview of the internal structure of the composite for a better understanding of all the features of the filler and the composite alike.

## 4. Gaps and Improvements

It was observed that biochar is an excellent filler that can be incorporated into polymer matrices to improve their overall properties. As it can be seen, a large emphasis has been given to the application of biochar in improving the mechanical and thermal properties of the filler. The application of biochar as an electrically conductive filler is still not quite explored. More parametric studies need to be developed to determine the impact of biochar on the mechanical and thermal properties of composites holistically. Rheological studies on the effect of biochar addition to the polymer were also not addressed in most of the studies discussed here. The study of rheology is important as it can have an impact on the processing and fabrication methods of the composite. Keeping in mind end use application of the composites, more focus on water absorption and dimensional stability is needed in the study. In the electrically conductive composite studies, emphasis on the temperature of pyrolysis and loading rate has been observed. Other fundamental properties like particle size of filler and morphology of filler have not yet been fully explored. Based on modelling studies on electrically conductive composites undertaken by Clingerman et al. (2003) [127], morphology of filler and particle size of filler have a significant effect on the electrical conductivity of the composite. There is a significant lack of modeling studies on the mechanical properties of the composites and development of such studies would help in further optimization of composite fabrication and properties. Another important factor ash content is not considered as relevant factor in the majority of the studies. One major factor that has not been addressed in any study is the effect of feedstock source on the biochar properties. Even though the end chemical composition and microscopic morphology of the biochar is fairly similar for biochar developed from any feedstock, it would be interesting to see if the nature of feedstock meaning the source, the type of source (grass, wood, fruit bunch, etc.,) has any effect on the final product. The moisture content, the amount of cellulose, lignin, hemicelluloses, etc., in the feedstock will greatly affect the end biochar produced from it [128]. The feedstock also dominates the ash content of biochar that can determine the properties of biochar and ultimately have an effect on the properties of the composites and its applications. Introduction of external coupling agents like compatibilizers has been done in studies and the effect of compatibilization has been observed on the mechanical properties of the composite. However, the effect of compatibilization of electrical conductivity of biochar filled composites is still yet to be explored. As most of these agents are polymeric in nature, it makes it interesting to see the effect of more polymeric material on the overall electrical conductivity of the composite. More studies can be based on development of biochar from waste-based sources like the studies conducted by Das, Sarmah, and Bhattacharyya (2016a) [14], Ketabchi et al. (2017) [129] and Poulose et al. (2018) [6]. The area of incorporation of biochar synergistically in polymer matrices to improve their properties is still being explored and there are several such gaps that can be addressed in future studies.

## 5. Conclusions

Biochar is an excellent additive to composites as most of the studies here have reported significant enhancement of composite properties with biochar addition. Biochar has properties like increased thermal stability, decay resistance, etc., that can make it better than the natural fillers like wood powder. The light weight of biochar makes it an attractive choice over conventional mineral fillers while considering weight reduction in automobiles. The ease of production of biochar from basically any lignocellulosic source makes it easy to procure and makes the entire process cost effective. So far, biochar has been successfully utilized in soil amendment and quality improvement. It has also shown its potential as a reinforcing filler in improvement of polymer mechanical and thermal properties opening up a plethora of application opportunities. Most of the studies addressed here have reported improvement in mechanical and thermal properties of polymer composites. With improved properties the applicability of the polymers is diversified. Composites can be developed using recycled polymers of biodegradable polymers to improve the sustainability of the entire lifecycle of the composites. There is great promise in the application of biochar as an electrically conductive filler and needs more exploration. However, it has been observed that biochar definitely acts as a conductive filler and has the potential to become a filler in conductive polymer composites. The ease of manufacturing and the freedom of customizing the properties as per need makes it very useful. Biochar is an emerging filler material in the field of material and polymer science. It has a lot of potential that needs harnessing, all is needed is focus on the right direction to make the most of it.

## Figures and Tables

**Table 1:** Fabrication methods and composition of biochar filled polymer composites.

Matrix	Filler	Filler Loading Rate (wt%)	Carbonization Temperature (°C)	Fabrication Method	References
Ultrahigh Molecular weight Polyethylene (UHMWPE)	Nano-bamboo charcoal	1, 3 and 9	1000	Compression Molding	[36]
Polypropylene	Maleic Anhydride grafted Polypropylene (MAPP) Biochar (1000 µm and 50 µm) Wood	0 and 4 24 0 and 30	900	Melt extrusion and injection molding	[28]
Polypropylene	Compatibilizer Biochar (<20 µm, 106–125 µm)	0, 2.5, 5, 7.5. Not reported	~630	Injection molding	[37]
Polypropylene	Poly Octene Ethylene copolymer (POE) Biochar	30 10 and 20	500 (LtBioC) 900 (HtBioC)	Melt compounding and injection molding	[38]
Polyamide 6, 10	Biochar (<63, 213–250, 426–500 µm)	20	500	Melt compounding and injection molding	[39]
Nylon 6	Biochar	20	500–900	Melt compounding and injection molding	[40]
Polypropylene	Bamboo particles Ultrafine bamboo char (UFBC)	5, 10, 15, 20, 25 and 30	Not reported	Melt compounding and injection molding	[10]
Polyethylene	Biochar	1 and 5	480	Solvent casting and melt mixing	[41]
Polypropylene	Biochar	15, 25, 30 and 35	900	Melt compounding and Injection molding	[5]
Polypropylene	Biochar Wood MAPP	24 Concentration not reported 0–3	900	Melt extrusion and injection molding	[42]
Polypropylene	Biochar Wood MAPP	6, 12, 18, 24, 30. 30 4	400 and 450	Melt extrusion and hot compression	[43]
Poly Lactic Acid (PLA)	Biochar	2, 6 and 10	Not reported	Solvent casting	[33]
Epoxy	Biochar Multiwalled Carbon Nanotubes (MWCNT)	2, 4 and 20	950 (BCHT)	Resin curing	[34]
Epoxy	Biochar	5, 10, 15 and 20	400, 600, 800 and 1000	Resin curing	[44]
UHMWPE	Biochar	60, 70 and 80	1100	Melt extrusion and hot compression	[45]
PLA	Biochar	1, 2.5, 5 and 7.5	700	Melt mixing and solvent casting	[46]
High Density Polyethylene (HDPE)	Biochar	30, 40, 50, 60, 70	500	Melt mixing and extrusion	[47]

**Table 2:** Mechanical properties of biochar filled polymer composites.

Polymer	Effective Biochar Loading Rate (%)	Young’s Modulus	Flexural Strength	Impact Strength	Contributing Factor	References
Polypropylene	35	3.82 GPa	58.26 MPa	Not reported	Loading rate of biochar	[5]
Polypropylene	24	37.76 MPa	75.12	~3 KJ/m^2^	Presence of MAPP and interlocking between polymer and filler	[42]
UHMWPE	9	388.4 MPa	Not reported	Not reported	Interfacial adhesion between matrix and filler	[36]
Polypropylene	Not reported	~5 GPa	~70 MPa	Not reported	Presence of MAPP and porous structure of biochar	[28]
Polypropylene	24	3.5 GPa	Not reported	Not reported	Porous structure of biochar facilitating mechanical interlocking	[59]
Polypropylene	20	~1500 MPa	Not reported	50 J/m	Higher carbonization temperature	[38]
Polyamide 6,10	20	~2.5 GPa	~110 MPa	~60 J/m	Particle size of biochar	[39]
Nylon 6	20	3.3 GPa	140 MPa	50 J/m	Carbonization temperature of biochar	[40]
Polypropylene	10	460.59 MPa	Not reported	~18 KJ/m^2^	Improved interfacial adhesion on biochar addition	[10]
Polyethylene	5	25 MPa	Not reported	Not reported	Lack of functional groups on biochar surface	[41]

**Table 3:** Electrical conductivity values reported for biochar filled polymer composites.

Polymer	Highest Biochar Loading Rate (wt%)	Carbonization Temperature (°C)	Fabrication Method	Variable Factor	Conductivity (S/cm)	References
Polyvinyl Alcohol (PVA)	10	Not reported	Solution casting	Loading rate of filler	1.833 × 10^−3^	[33]
Ultra-High Molecular Weight Polyethylene (UHMWPE)	80	1100	Melt extrusion and hot compression	Loading rate of filler	107.6	[45]
Epoxy	20	1000	Resin curing	Carbonization temperature	2.02	[44]
Epoxy	20	950	Resin curing	Loading rate of filler	~0.75	[34]

## Data Availability

The data presented in this study are available on request from the corresponding author.
